# Recurrent ischemic stroke: Incidence, predictors, and impact on mortality

**DOI:** 10.1111/ane.13093

**Published:** 2019-04-11

**Authors:** Andrej Netland Khanevski, Anna Therese Bjerkreim, Vojtech Novotny, Halvor Næss, Lars Thomassen, Nicola Logallo, Christopher E. Kvistad, the NOR‐STROKE study group

**Affiliations:** ^1^ Department of Clinical Medicine University of Bergen Bergen Norway; ^2^ Department of Neurology Haukeland University Hospital Bergen Norway; ^3^ Norwegian Health Association Oslo Norway; ^4^ Centre for Age‐Related Medicine Stavanger University Hospital Stavanger Norway; ^5^ Department of Neurosurgery Haukeland University Hospital Bergen Norway

**Keywords:** cerebrovascular disease, neuroepidemiology, stroke etiology, stroke recurrence

## Abstract

**Background and purpose:**

Recurrent ischemic stroke (IS) or TIA is frequent with a considerable variation in incidence and mortality across populations. Current data on stroke recurrence and mortality are useful to examine trends, risk factors, and treatment effects. In this study, we calculated the incidence of recurrent IS or TIA in a hospital‐based stroke population in Western Norway, investigated recurrence factors, and estimated the effect of recurrence on all‐cause mortality.

**Methods:**

This prospective cohort study registered recurrence and mortality among 1872 IS and TIA survivors admitted to the stroke unit at Haukeland University Hospital between July 2007 and December 2013. Recurrence and death until September 1, 2016, were identified by medical chart review. Cumulative incidences of recurrence were estimated with a competing risks Cox model. Multivariate Cox models were used to examine recurrence factors and mortality.

**Results:**

During follow‐up, 220 patients had 277 recurrent IS or TIAs. The cumulative recurrence rate was 5.4% at 1 year, 11.3% at 5 years, and 14.2% at the end of follow‐up. Hypertension (HR = 1.65, 95% CI 1.21‐2.25), prior symptomatic stroke (HR = 1.63, 95% CI 1.18‐2.24), chronic infarcts on MRI (HR = 1.48, 95% CI 1.10‐1.99), and age (HR 1.02/year, 95% CI 1.00‐1.03) were independently associated with recurrence. A total of 668 (35.7%) patients died during follow‐up. Recurrence significantly increased the all‐cause mortality (HR = 2.55, 95% CI 2.04‐3.18).

**Conclusions:**

The risk of recurrent IS stroke or TIA was modest in our population and was associated with previously established risk factors. Recurrence more than doubled the all‐cause mortality.

## 1 INTRODUCTION

The incidences of stroke and post‐stroke mortality have declined heavily in high‐income countries in the last 50 years, mainly because of changes in cardiovascular risk factors and improvements in acute stroke treatment.^1^ Despite progress and declining trends, recurrent stroke is still frequent.^2^, ^3^ Studies demonstrate varying recurrence rates, ranging from 7%‐20% at 1 year to 16%‐35% at 5 years.^4^, ^5^ Recurrent ischemic stroke (IS) has been associated with increased mortality and functional dependence,^6^, ^7^ but this remains insufficiently explored. The primary goal of secondary prevention strategies after IS or transient ischemic attack (TIA) is to reduce the risk of recurrent stroke, and information on stroke recurrence and survival is useful to assess the effect of secondary prevention and risk factors for recurrence and death.

In this study, we aimed to investigate the cumulative incidence of recurrent IS or TIA in a Norwegian hospital‐based stroke population, as well as investigate factors associated with recurrence. We also wanted to assess the all‐cause mortality after IS or TIA and the influence of recurrence on mortality.

## 2 MATERIALS AND METHODS

In this hospital‐based prospective cohort, all patients older than 18 years admitted with IS or TIA to the Stroke Unit at the Department of Neurology, Haukeland University Hospital, between July 1, 2007, and December 31, 2013, were prospectively registered in the Bergen NORSTROKE Registry. Approximately 275 000 people are living in the area served by our stroke unit. For this analysis, we only included patients that lived inside this area at the time of the index IS or TIA. Patients that moved outside of the defined geographic area before the end of data collection, and patients that died during index admission or were discharged to palliative care, were excluded from the analysis. Patients from our area admitted to other hospitals with index stroke were not included in the study.

Ischemic stroke was defined as an episode of neurologic deficit lasting >24 hours or clinical symptoms where magnetic resonance imaging (MRI) or computed tomography (CT) showed infarctions related to the clinical findings.^8^ TIA was defined as a clinical diagnosis of transient focal cerebral dysfunction lasting <24 hours with no objective evidence of brain infarction on brain imaging. Chronic infarctions were defined as MRI findings of focal hyperintensity lesions on T2 sequences and low signal intensity in the presence of gliosis and/or cystic encephalomalacia on FLAIR sequences.^9^ Differentiation from perivascular spaces and other lesions was made by a neuroradiologist or experienced stroke clinician (HN). Brain imaging (CT and/or MRI), carotid ultrasound, and 24‐hour electrocardiographic monitoring were obtained during index admission. The Trial of Org 10172 in Acute Stroke Treatment (TOAST) criteria were used for etiology classification.^10^ Clinical symptoms were classified with the Oxfordshire Community Stroke Project Classification (OCSP).^11^ Clinical characteristics, NIHSS, mRS, comorbidity, and medical history including previous IS or TIA were registered during index admission.

Definition of recurrent IS or TIA was the same as for the index event. Clinical recurrence was identified by review of electronic medical records from all ten hospitals under the Western Norway Regional Health Authority. Information on death was collected electronically from the Norwegian National Registry. Patients were followed until September 1, 2016. Data collection was performed mainly during 2017. We did not include intracerebral hemorrhage or subarachnoid hemorrhage as recurrent events. The study was approved by the Western Regional Ethics Committee with written informed consent obtained from all patients or their legally authorized representatives.

## 3 STATISTICS

Univariate analyses were performed using the chi‐squared test for categorical variables and Student's *t* test or Mann‐Whitney's *U* test for continuous variables. Categorical variables are presented as N (%), and continuous variables are expressed as mean ± standard deviation (SD) or as median with interquartile range (IQR) if skewed. Cumulative incidence of recurrence was calculated with the cumulative incidence function (CIF) after applying the method of Fine and Gray by treating death as a competing risk.^12^ Kaplan‐Meier method was used to estimate the incidence of all‐cause mortality. Factors independently associated with IS or TIA recurrence were assessed with backward stepwise Cox regression by analysis of variables that turned out significant (*P* < 0.05) in the univariate analyses. The effect of recurrence on all‐cause mortality was analyzed by creating a Cox regression model with recurrence as a time‐dependent covariate. Known mortality‐associated factors such as age, smoking, stroke severity, and risk factors for stroke were included in the model. We analyzed the data with Stata/SE 15.1 (Stata Corp LLC,).

## 4 RESULTS

During the inclusion period, 1988 patients were diagnosed with IS or TIA, of whom 116 died during the index admission or were discharged to palliative care (5.8%). The final cohort consisted of 1872 patients with index IS (n = 1666) or TIA (n = 206). The mean age was 73.2 years (range 18‐99 years) and 837 were women (44.7%). MRI was performed in 1629 patients (87.1%) during the index hospitalization. A total of 243 patients (13.0%) had a history of previous IS, and 144 (7%) had a history of previous TIA.

At index admission, 714 (38.1%) and 158 (8.4) patients were already on antiplatelet agents and anticoagulation agents, respectively. At index discharge, the indicated drugs were prescribed in 1228 (65.6%) and 630 (33.7%) patients. The number of patients on lipid‐lowering drugs, antihypertensive drugs, and antidiabetic drugs at index admission was 537 (28.7%), 886 (47.3%), and 206 (11.0%), respectively. At index discharge, that increased to 1331 (71.1%), 1178 (62.9%), and 247 (13.2%) patients. Only 0.8% of patients (n = 14) were not assigned any secondary prevention, mainly because of contraindications. Cardioembolism was the most frequent determined etiology (n = 604, 32.3%), followed by large‐artery atherosclerosis (N = 244, 13.0%), small vessel occlusion (N = 205, 11.0%), and stroke of other determined etiology (N = 32, 1.7%). In 787 patients (42.1%), an etiology could not be determined. Partial anterior circulation infarct (PACI) was the most common clinical Oxfordshire subtype at index (n = 882, 47.3%).

**Figure 1:**
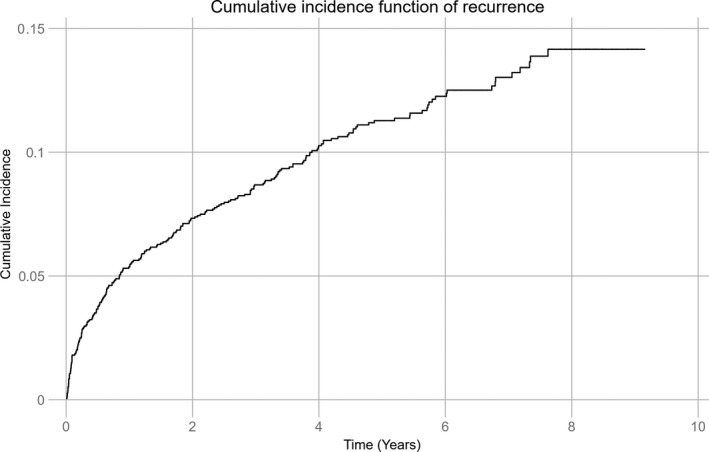
Cumulative incidence of first recurrence of ischemic stroke or TIA

During a mean follow‐up of 5.6 years (median 5.5 years, max. 9.2 years, 8172.8 person‐years), 220 (11.7%) patients experienced 241 (87%) recurrent IS and 36 (13%) TIA. One recurrent event was observed in 175 patients, two in 36 patients, three in six patients, and four in three patients. First recurrence was IS in 195 (88.6%) patients and TIA in 25 (11.4%) patients. Median time to the first recurrence was 436.5 days. Figure 1 demonstrates the cumulative incidence function adjusted for age (CIF) of recurrent IS and TIA. The total cumulative recurrence rate adjusted for age during follow‐up was 14.2%. After 1 year and 5 years, recurrence was observed in 101 (5.4%) and 201 (10.7%) patients, respectively, resulting in age‐adjusted cumulative recurrence of 5.4% and 11.3% at 1 year and 5 years, respectively. The total incidence rate per 1000 person‐years during follow‐up was 34.0 (95% CI 30.2‐38.2), being the highest the first year with 67.8 recurrences (95% CI 56.6‐81.2) per 1000 person‐years.

Baseline characteristics of the cohort stratified by recurrence status at the end of follow‐up are presented in Table 1. Patients with recurrence had a significantly lower discharge mRS score, more frequently a history of previous IS or TIA as well as chronic infarcts seen on MRI, a history of hypertension, and was more frequently prescribed antiplatelet agents and antihypertensive drugs at index admission.

**Table 1:** Baseline characteristics at index stroke (N, %)

	No recurrence	Recurrence	
	N = 1652	N = 220	*P*
Age (y), mean ± SD	73.0 ± 13.8	75.0 ± 11.3	0.07
Male sex	917 (55.5)	118 (53.6)	0.60
mRS score at index discharge, median (IQR)	2 (1, 3)	1 (0, 3)	0.03
NIHSS score at admission, median (IQR)	2 (1, 5)	2 (0, 5)	0.20
Stroke diagnosis at index			
Ischemic stroke	1468 (88.9)	198 (90.0)	0.61
TIA	184 (11.1)	22 (10.0)	0.61
TOAST subtype at index			
Large‐artery atherosclerosis	207 (12.5)	37 (16.8)	0.08
Cardioembolism	537 (32.5)	67 (30.4)	0.54
Small vessel occlusion	187 (11.3)	18 (8.2)	0.16
Other determined etiology	27 (1.6)	5 (2.3)	0.49
Undetermined etiology	694 (42.0)	93 (42.3)	0.94
OCSP classification			
TACI	164 (10.0)	15 (6.9)	0.15
PACI	771 (48.9)	111 (50.9)	0.26
POCI	322 (19.6)	46 (21.1)	0.60
LACI	388 (23.6)	46 (21.1)	0.42
Comorbidities prior to index event			
Prior ischemic stroke	199 (12.1)	44 (20.0)	<0.01
Prior TIA	116 (7.0)	28 (12.7)	<0.01
Peripheral artery disease	117 (7.1)	19 (8.6)	0.40
Coronary artery disease	367 (22.2)	51 (23.2)	0.75
Diabetes mellitus	240 (14.5)	41 (18.6)	0.11
Atrial fibrillation	476 (28.8)	67 (30.5)	0.61
Hypertension	902 (54.6)	150 (68.2)	<0.01
Ever‐smoker	956 (57.8)	130 (59.1)	0.73
Length of stay, median (IQR)	6 (3, 10)	5 (3, 8)	0.17
Type of prophylaxis at index discharge			
Antiplatelet agent	1069 (64.7)	159 (72.2)	0.03
Anticoagulation agent	570 (34.5)	60 (27.3)	0.03
No prophylaxis	13 (0.8)	1 (0.5)	0.45
Silent infarcts seen on MRI at index	466 (32.5)	85 (43.8)	<0.01
Other medications at index discharge			
Statins and other lipid‐lowering drugs	1174 (71.1)	157 (71.4)	0.93
Antihypertensive drugs	1025 (62.1)	153 (69.6)	0.03
Antidiabetic drugs	209 (12.5)	38 (17.3)	0.06

Abbreviations: IQR, interquartile range; LACI, lacunar infarct; mRS, modified Rankin Scale; NIHSS, National Institutes of Health Stroke Scale; PACI, partial anterior circulation infarct; POCI, posterior circulation infarct; TACI, total anterior circulation infarct.

Table 2 shows factors independently associated with recurrence from the final Cox regression model. History of hypertension at index admission was the strongest independent risk factor, with a 65% increased risk for recurrence. Previous symptomatic cerebrovascular disease was associated with a 63% higher risk of recurrence, while chronic infarcts seen on MRI at index admission increased the risk with 48%. Older age increased the risk of recurrence with 2% per year. There were no differences between patients treated with antiplatelet agents or anticoagulants.

**Table 2:** Factors associated with recurrence after IS or TIA, (n = 1621)

	HR	95% CI	P
Age, y	1.02	1.00‐1.03	0.01
Female	0.99	0.74‐1.33	0.96
mRS score	0.91	0.82‐1.01	0.07
History of IS or TIA	1.63	1.18‐2.24	<0.01
History of hypertension	1.65	1.21‐2.25	<0.01
Chronic infarcts seen on MRI at index	1.48	1.10‐1.99	0.01

Abbreviations: HR, hazard ratio; mRS, modified Rankin Scale; NIHSS, National Institutes of Health Stroke Scale.

**Figure 2:**
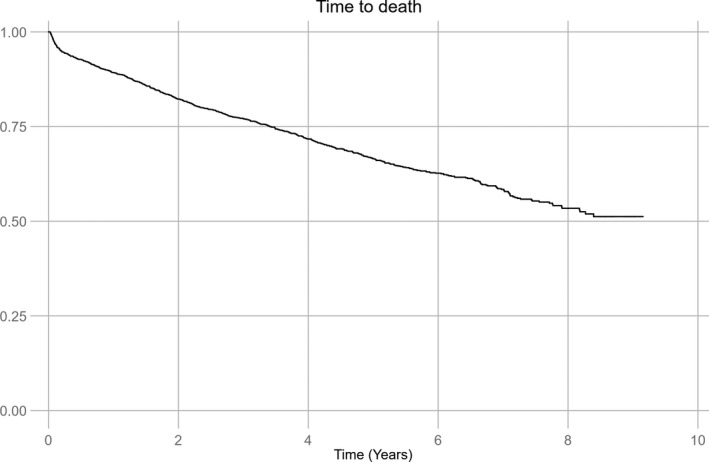
Time do death after index ischemic stroke or TIA

A total of 668 (35.7%) patients died during follow‐up. Of the 220 patients with recurrence, 96 (43.6%) died, whereas 572 (34.6%) of the 1652 patients without recurrence died (*P* < 0.01). Kaplan‐Meier estimates representing the risk of all‐cause death after stroke are demonstrated in Figure 2. Total cumulative incidence of all‐cause death during follow‐up was 51.2%, varying from 47.4% in patients with no recurrence to 57.8% in patients with recurrence. The cumulative risk of all‐cause death at 1 and 5 years was 10.7% and 33.5%, respectively. Factors independently associated with mortality are presented in Table 3. Recurrent IS or TIA was the strongest risk factor for death, with a 2.5‐fold increase in the all‐cause mortality adjusted for known factors independently increasing the risk of death such as smoking, diabetes, coronary artery disease, older age, and stroke severity at discharge. When we excluded the 25 patients with TIA as the first recurrent event from the mortality analysis, a 2.6‐fold increase in the risk of death in patients with recurrent IS was found. Patients treated with statins for high cholesterol had a lower risk of death.

**Table 3:** Impact of recurrence on all‐cause mortality after IS or TIA, (n = 1854)

	HR	95% CI	*P*
Recurrent stroke	2.55	2.04‐3.18	<0.01
Age, y	1.09	1.08‐1.10	<0.01
Female	0.85	0.72‐1.00	0.06
NIHSS at discharge	1.08	1.07‐1.10	<0.01
History of coronary artery disease	1.22	1.03‐1.45	0.02
History of diabetes	1.48	1.21‐1.80	<0.01
Previous or current smoking at index	1.47	1.24‐1.74	<0.01
Statins and other lipid‐lowering drugs at index discharge	0.81	0.69‐0.96	0.02

Abbreviations: HR, hazard ratio; NIHSS, National Institutes of Health Stroke Scale.

## 5 DISCUSSION/CONCLUSION

In this prospective cohort study, the cumulative incidence of clinical recurrent IS or TIA was 5.4% at 1 year and 11.3% at 5 years. This is considerably lower compared with a meta‐analysis from 2011, which estimated a pooled cumulative risk of 11.1% at 1 year and 26.4% at 5 years.^4^ One explanation for this difference could be the declining incidences of stroke recurrence, which have been observed in studies on temporal trends of recurrence.^2^, ^3^ Furthermore, optimal use of secondary prevention strategies has shown a significant reduction in the absolute risk of recurrence.^13^ Almost all patients in our study were prescribed antiplatelet or anticoagulant treatment, and the use of statins and antihypertensive treatment was high compared to other studies.^14^ Finally, differences in populations, methods, and use of statistical analyses in studies on recurrence make comparison between them difficult. The Kaplan‐Meier survival analysis may overestimate the cumulative incidence of recurrence since death is not treated as a competing risk.^15^ Recent studies adjusting for competing risk of death have reported 1‐ and 5‐year incidences of recurrence of 3.6%‐7.7% and 10.1%‐16.8%.^16^, ^17^, ^18^, ^19^ Our results are thus supporting newer data from several other populations.

Among the factors associated with recurrence in our study, previous hypertension at index stroke was the strongest one. Hypertension is usually considered the single most important risk factor for first stroke, but its role in the risk of recurrence is uncertain. Some studies have reported this association,^7^, ^20^ while other studies have found diabetes, coronary heart disease, and atrial fibrillation as the strongest risk factors.^21^, ^22^ Neither of these were independently associated with recurrence in our study and did not differ between patients with or without recurrence (Table 1). No single factor has been consistently associated with recurrent IS or TIA.^21^ Case mix, changes in risk factor profiles, and risk factor control may affect the significance of independent factors over time and influence the results of individual studies.

In our cohort, we studied a general hospital‐based stroke population for recurrence and included patients with a history of IS or TIA thus making the results relevant for clinical practice. Both previous IS or TIA and chronic infarcts on MRI were independently associated with a higher risk of recurrent stroke. Although some studies use the concept of “first stroke” as index event, we chose to include patients with prior stroke at index IS or TIA in this study. While 19.2% of the patients in this study had a history of prior clinical IS or TIA, almost twice as many (33.8%) had chronic infarcts visible at MRI during index IS or TIA. In a recent meta‐analysis, chronic brain infarction at index IS or TIA doubled the risk of recurrent stroke, which is similar to our results.^23^

Recurrent ischemic stroke or TIA was a strong independent factor doubling the mortality risk in our cohort. Despite the overall high incidence of recurrent stroke, few studies have investigated the impact of recurrence on all‐cause mortality. Studies have reported unadjusted estimates ranging from a 2‐fold increase in mortality to a 17‐fold increase.^7^, ^24^ However, two other studies using similar statistical approaches as in our study reported adjusted relative mortality risks of 2.7 and 3.0 after recurrence,^6^, ^25^ which are more similar to our findings. The mechanisms behind how recurrence affects mortality are not well studied. Stroke severity is considered the most significant factor influencing short‐ and long‐term outcome after stroke,^26^ and when recurrent stroke adds to existing neurological deficit, patients may be more prone to complications and a worse outcome. One study found that a contralateral recurrent stroke led to more severe functional disability than patients with ipsilateral recurrence, which reduces the brain's ability to compensate.^7^

Our study has limitations. First, we may have underestimated the true incidence of recurrence, as recurrent events were identified by review of medical records, and thus patients had to be admitted to a hospital within our region to be included as a recurrent event. Furthermore, we have no data on drug compliance and scarce data on cause of death, which limits the interpretation of factors affecting mortality. Finally, the findings from our Caucasian population with mostly mild IS or TIA and high rates of secondary prevention may not be transferable to other demographics.

The main strength of this study is that we have obtained high‐quality 1‐ and 5‐year recurrence and mortality data in a hospital‐based stroke population. A predefined investigation protocol and the use of electronic medical records for identification of recurrent IS or TIA provide precise and accurate clinical data on both index event and on the recurrent episode as compared to studies using administrative data. All ischemic stroke cases were confirmed with imaging studies. On this background, we conclude that the risk of recurrent IS or TIA is modest in our population and that recurrent stroke has a high negative impact on all‐cause mortality.

## CONFLICT OF INTEREST

None.

## DATA AVAILABILITY

The anonymized data that support the findings of this study are available on request from the first author, ANK. The data are not publicly available due to their containing information that could compromise the privacy of research participants.
